# Economic policy uncertainty and the US stock market trading: non-ARDL evidence

**DOI:** 10.1186/s43093-022-00150-8

**Published:** 2022-09-09

**Authors:** Bakhtiar Javaheri, Fateh habibi, Ramin Amani

**Affiliations:** Department of Economics, University of Kurdistan, Sanandaj, P.O. Box: 416, Iran

**Keywords:** Economic policy uncertainty, Economic factors, Stock market, Non-ARDL, United States, C01, C32, G10

## Abstract

The present study investigates the impact of economic policy uncertainty, and economic factors on the stock market index in the USA using Non-ARDL and Quantile models. The findings reveal that declining economic and economic-political factors will increase the stock market index in the US. The results indicate that the effect of inflation and GDP variables follows a nonlinear pattern. Similar results using quantitative regression showed asymmetric impacts of inflation and GDP on stock market transactions.

## Introduction

The impact of global events on stock prices, especially after the significant increase and decrease in the stock market in recent years, has attracted financial economists. During civil and political turbulence, stock markets usually face enhanced fluctuations because important political events indicate a potential change in politics that may change the market value [9]. Stock market fluctuation is significantly important to policymakers and portfolio directors at the time of visualizing the future corporate health and investment outlooks [4].

Baker et al. [3] developed an EPU index by dividing the papers related to the uncertainty of policy by the entire paper. This index is a criterion for the next studies. Global EPU overflows are more important because of the increasing trend after the 2007–2008 global financial crisis. After the Brexit and Trump election, the EPU index experienced the highest level. As a result of economic globalization, the stock market in a country is affected by the country’s EPU as well as uncertainty in other powerful countries [14, 27]. Therefore, the identification of the effect of significant EPU indexes on stock market fluctuation is very important.

While Ewing and Malik [18] emphasize the important role of energy costs, particularly oil prices, over the stock markets, several authors emphasized the significance of overflow from developed countries, and particularly the US market, to emerging stock markets.

The coronavirus in 2019 (COVID-19) has created not only a disruption to everyday life but also a crisis in the stock market all around the world. The damage caused by this crisis may be more severe than the previous crises. Most governments adopt a wide range of lockdown policies, their economic activities have become very limited, and eventually, COVID-19 may lead to widespread unemployment and commercial failures [51, 52]. The COVID-19 epidemic had a profound effect on the financial markets [4]. Meanwhile, the prevalence of COVID-19 has also led to a change in other macroeconomic variables’ trends.

The present paper aims to explain the relative importance of EPU indicators in the US stock markets. Consequently, participants and policymakers will be able to identify the source of stock market risk for policymaking. The second aim is to expand the knowledge of the asymmetric connection between the EPU and the stock market risk. This is helpful for portfolio selection, risk management, and financial stability research. The third aim is to present patterns of asymmetric and nonlinear behavioral impact of post-political shock market fluctuations in a particular period.

Although advanced linear models for examining the effective factors of stock market transactions have had good results in the short-term and medium-term, they affected the stock market as a result of asymmetric and nonlinear behaviors. For this purpose, NARDL and Quantile regression will be used in this study. The reason for selecting the US stock market is that its stock market is significantly important for systematic risk.

The rest of this paper is organized as follows: “Literature review” section provides a brief overview of the related literature on the subject. “Data and methodology” section explains the data and methodology. “Results and discussion” section shows the results and discussions. “Conclusion” Section concludes the paper and presents the conclusion.

## Literature review

The economic policy uncertainty at high levels has a negative impact on the macro economy and the stock market [50]. The impact of economic policy uncertainty on the stock market can be explained by both supply and demand side channels [30]. On the demand side, with the increase in economic policy uncertainty, companies are expected to reduce and stop investment demand, and this itself can have a negative impact on the stock market. On the supply side, the increase in economic policy uncertainty can increase the cost of hiring labor and lead to a negative impact on companies and the stock market [13, 15]. Several other studies have also obtained key evidence about the negative impact of economic policy uncertainty on the stock market [2, 6, 10, 33, 35, 46, 48].

Moreover, economic policy uncertainty refers to policies that determine the rules of the game for economic factors Baker et al. [3]. The economic policy uncertainty through 4 major channels can affect the prices of various assets in the stock market. First, economic policy uncertainty may change or delay important decisions made by firms and other economies such as employment, investment, consumption, and savings. Second, economic policy uncertainty may exacerbate disinvestment and economic contraction by affecting financing and production costs, increasing both supply and demand channels [25]. Third, economic policy uncertainty may reduce prosperity in financial markets by increasing risk [41]. Fourth, economic policy uncertainty can affect financial markets by influencing interest rates and inflation rates [42].

The impact that global events have had on stock prices, especially after the significant increase and decrease in the stock market in recent years, has attracted the interest of financial economists. In the literature, variations in internal factors, such as economic, political, financial, and global factors, may change the stock market in emerging markets. Chau et al. [9] and Mnif [36] argued that stock markets may have a negative effect on domestic political vulnerability. Lobo [34] investigates markets in the US midterm elections in 1998 after the disclosure of political scandals and concludes that there is a lot of insecurity among investors.

In addition, Perotti and Oijen [43] conduct research in emerging markets to determine the impact of political shocks on the stock exchange. Their findings indicate that when the political risk increases or decreases, significant changes happen in excess returns, showing that political risk is a significant factor in pricing at some point in stock returns. Jackson [24] reviews the global economy after September 11, one of the biggest events of the twenty-first century, showing that although the attack occurred in the United States, the global markets have been affected. Chesney et al. [11] examine the effects of 77 terrorist attacks that have taken place in 25 countries on the global economy and conclude that most of these events have a negative impact on financial markets.

Many authors and researchers examine the impact of EPU on various areas, such as EPU reduction returns [10], real loan growth [5], and increasing unemployment rate [8]. Liu and Zhang [33] use the framework of heterogeneous auto-regressive realized volatility (HAR) provided by Corsi [19] to apply EPU’s prediction ability in the US stock market. The evidence shows that the EPU index highly enhances the model’s prediction performance.

Dong et al. [16] check the alteration of affiliated structures between the stock markets and economic factors during the COVID-19 epidemic. Obtained results indicate that the stock market is mostly affected by economic factors during the COVID-19 outbreak.

He [23] studies the impact of the asymmetric overflow of significant economic policy uncertainty (EPU) on the S&P500 index. The results show that the S&P500 index fluctuations in the net overflow are important EPU indicators. The Japanese EPU has the strongest overflow in the US stock exchange, while EPU from the UK has a limited effect.

Li et al. [31] examine the effect of global economic policy uncertainty (GEPU) on Chinese stock market fluctuations. According to the results, the rise and fall in the GEPU can lead to significant fluctuations in the stock market for China. In addition, the directional GEPU compared to an increase in the prediction accuracy can provide more useful information. Also, empirical results show that directional GEPU is more influential in forecasting Chinese stock market fluctuations when GPU and EPU increase in the same month. Kirikkaleli [26] examines the impact of internal factors—economic, financial, and political risk—and external factors—universal economic policy uncertainty—on the stock market index in Taiwan. These findings show that a mixture of internal and external risk factors has a long-term impact on the stock market index. Additionally, the decline in economic, political, and financial risks leads to an increase in the stock market index in Taiwan.

In another study, Chiang [12] examines the EPU, risk, and stock returns using the G7, and reports that delayed EPU innovations significantly affect the conditional variance. Liu and Zhang [33] examine EPU’s effects on future fluctuations based on multi-factor insights, indicating the increase of prediction accuracy by the EPU index.

Li and Giles [32] find significant one-way shock and unilateral fluctuations from the US market to emerging Asian markets. Chau et al. [9] study the effect of political uncertainty on major stock market fluctuations in the MENA region. The results find a rise in the fluctuations of Islamic indicators during political turbulences, while the uprisings had a slight or no significant impact on the fluctuations in ordinary markets. Similar results are not observed for criteria indicators, showing that changes are due to political tensions.

## Data and methodology

This study empirically used time series variables consisting of annual data from 1990 to 2019. As shown in Table 1, the variables, including Stock Market Traded (current US$), GDP (constant 2010 US$), Inflation Rate (annual %), Interest Rate, Unemployment Rate (percentage of the total labor force), Government Debt (% of GDP) and Real effective exchange rate index are obtained from the World Bank, while the Economic Policy Uncertainty index is obtained from the Economic Policy Uncertainty Index’ web site.

**Table 1:** Definition of variables and summary of data sources

Variables	Definition of variables	Sources
SM	Stocks traded, total value (current US$)	World bank
GDP	GDP (constant 2010 US$)	World bank
INR	Real interest rate (%)	World bank
INF	Inflation, consumer prices (annual %)	
UM	Unemployment, total (% of the total labor force)	World bank
GD	Central government debt, total (% of GDP)	World bank
ER	Real effective exchange rate index (2010 = 100)	World bank
EPU	Economic Policy Uncertainty	www.policyuncertainty.com/

Source: Research finding

To construct an Economic Policy Uncertainty (EPU) Index, the process is as follows: First, re-normalize each national EPU index to a mean of 100 from 1990 (or first-year) to 2019. Second, impute missing values by using a regression-based method. This step yields a balanced panel of monthly EPU index values for the U.S. Third, compute the EPU Index value for each month as the GDP-weighted average of the EPU index values, using GDP data from the IMF’s World Economic Outlook Database. (http://www.policyuncertainty.com) construct two versions of the EPU Index—one based on current-price GDP measures, and one based on PPP-adjusted GDP. The automated text-search results of the electronic archives of 11 national and international newspapers are reflected by the EPU index; these newspapers include Financial Times, The Boston Globe, Chicago Tribune, The Globe and Mail, The Daily Telegraph, The Guardian, The New York Times, The Times, Los Angeles Times, The Washington Post, and The Wall Street Journal. Then the index is normalized to the average value of 100 in the 1990–2019 period (policyuncertainty.com). EPU was constructed based on a text-search algorithm from the leading national newspapers. The EPU index includes many words in the newspaper articles, such as “economy” or “economic”; “uncertain” or “uncertainty”.

Data on the U.S. EPU is available on a monthly basis, which was converted to annual using the averaging method as follows:

${\text{EPU}}_{t} = \frac{{{\text{EPU}}_{m1} + {\text{EPU}}_{m2} + {\text{EPU}}_{m3} + \cdots + {\text{EPU}}_{m12} }}{12}$, where ${\text{EPU}}_{t}$ is economic policy uncertainty index of the considered year and ${\text{EPU}}_{m1} + {\text{EPU}}_{m2} + {\text{EPU}}_{m3} + \cdots + {\text{EPU}}_{m12}$ is economic policy uncertainty index of the first month to the last month.

**Fig. 1:**
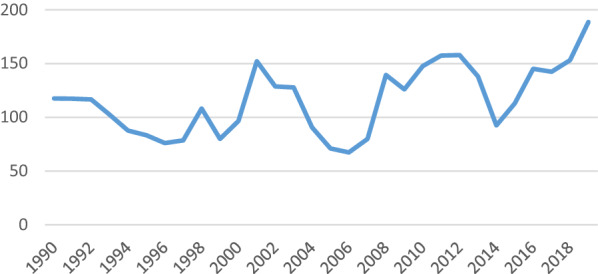
Time Trend of U.S. EPU. *Source* Research finding

Furthermore, Table 2 shows the descriptive statistics. Regarding Stock market traded and GDP, the maximum values are 4.72E+13 and 1.83E+13 US$, respectively, and the minimum values are 2.03E+12 and 8.99E+12 US$, respectively. The minimum values of the Interest and Inflation rates include 1.13% and − 0.35%, respectively, whereas the maximum values are 7.14% and 5.39%, respectively. The average values of US government debt and unemployment rate from 1990 to 2019 are 15.16% of GDP (Gross Domestic Product) and 5.84% of the labor force, respectively. Regarding the exchange rate index and economic policy uncertainty index, the minimum and maximum values are 95, 67.13, 126.22, and 188.69, respectively. Furthermore, Fig. 1 displays the time trend U.S. EPU index.

**Table 2:** Descriptive statistics

Variables	Obs	Mean	Std. dev	Min	Max
SM	30	2.37E+13	1.47E+13	2.03E+12	4.72E+13
GDP	30	1.36E+13	2.81E+12	8.99E+12	1.83E+13
INR	30	3.77	1.93	1.13	7.14
INF	30	2.44	1.13	− 0.35	5.39
UM	30	5.84	1.59	3.67	9.63
GD	30	65.94	28.21	15.16	128.17
ER	30	108.52	8.25	95.00	126.22
EPU	30	116.03	31.74	67.13	188.69

Source: Research finding

The EPU index is sharply increased due to events, such as the Asian Financial Crisis, the 9/11 terrorist attacks, the U.S.-led invasion of Iraq in 2003, the Global Financial Crisis in 2008–2009, the European immigration crisis, concerns about the Chinese economy in late 2015, the Brexit referendum in 2016, and tensions between the US and China in 2018 [3, 21].

In examining the effect of GDP, Inflation, Interest Rate, Unemployment Rate, Government Debt, and Real effective exchange rate index and Economic Policy Uncertainty on Stock Market Traded of the United States, the following equation (Eq. 1) is used:

1 $$\begin{aligned} {\text{SM}}_{t} = & \,\alpha_{0} + \alpha_{1} {\text{GDP}}_{t} + \alpha_{2} {\text{INR}}_{t} + \alpha_{3} {\text{INF}}_{t} + \alpha_{4} {\text{UM}}_{t} \\ & \, + \alpha_{5} {\text{GD}}_{t} + \alpha_{6} {\text{ER}}_{t} + \alpha_{7} {\text{EPU}}_{t} + \varepsilon_{t} \\ \end{aligned}$$

where ${\text{SM}}_{t}$ is the stock market traded, ${\text{GDP}}_{t}$, the gross domestic production, ${\text{INR}}_{t}$, the real interest rate, ${\text{INF}}_{t}$, the inflation rate, ${\text{UM}}_{t}$, the unemployment rate, ${\text{GD}}_{t}$, the government rate, ${\text{ER}}_{t}$, the real effective exchange rate index, and ${\text{EPU}}_{t}$, the economic policy uncertainty index. Also, $\varepsilon_{t}$ is the residual term. The empirical model in the present study is constructed according to the model of Kirikkaleli [26] to explore the impact of internal factors—financial, political, and economic risks, external factors—global, economic, and political uncertainties, GDP and exchange rate on the stock market index in Taiwan in the period 1997Q1–2015Q2. However, Kirikkaleli [26] did not take into account the main economic factor in its study. Although, the studies of Abdelkafi [1], Ozer and Karagol [40], Próchniak and Witkowski [47], and Murgia [37] clearly emphasized the significance of the main economic factors (GDP, INR, INF, UM, GD and ER) and the uncertainties (EPU) on stock market trading.

In the present study, the first aim is to analyze the integration order of SM, GDP, INR, INF, UM, GD, ER, and EPU variables through the Ng—Perron Unit root test, developed by Ng and Perron [39]. The test contains four test statistics: MZa, MZt, MSB, and MPT. To design the preliminary version of the Phillips and Peron unit root test, Ng and Perron [39] used the GLS ERS trend reduction method.

Long-term co-integration bonding is on the account of models presented in Eq. 1 and is diagnosed using the ARDL bound test of Pesaran et al. [44, 45] after identifying time series variables as constant. The ARDL bounds test method is to estimate an unlimited error correction model (UECM). This test is better than the traditional co-integration methods. The bound test presents more accurate estimation results than traditional co-integration tests, especially for small sample sizes. Furthermore, unbiased estimations are conducted for the long-run model [22]. The bound test method is mostly dynamic, not restrictive, allowing it to be used whenever the model variables are integrated into one and zero—I (1) and I (0). Also, the bound testing prevents the indignity problem, particularly, if there is an endogenous repressor in the model, then F-tests, test statistics, and unbiased long-term estimations are valid yet. The statistical value of F was used by Pesaran et al. [44] to estimate the co-integration of Eq. (1), if the F-statistic is higher than the upper and lower bound critical values. It, therefore, confirms that the null hypothesis of no long-run relationship among variables stands rejected. The equation of the model of the long-term ARDL model is indicated as follows (Eq. 2):

2 $$\begin{aligned} {\text{SM}}_{t} = & \,\alpha_{0} + \mathop \sum \limits_{i = 1}^{m} \alpha_{1i} {\text{SM}}_{t - i} + \mathop \sum \limits_{i = 0}^{n} \alpha_{2i} {\text{GDP}}_{t - i} + \mathop \sum \limits_{i = 0}^{n} \alpha_{3i} {\text{INR}}_{t - i} \\ & \, + \mathop \sum \limits_{i = 0}^{n} \alpha_{4i} {\text{INF}}_{t - i} + \mathop \sum \limits_{i = 1}^{n} \alpha_{5i} {\text{UM}}_{t - i} + \mathop \sum \limits_{i = 1}^{n} \alpha_{6i} {\text{GD}}_{t - i} \\ & \, + \mathop \sum \limits_{i = 1}^{n} \alpha_{7i} {\text{ER}}_{t - i} + \mathop \sum \limits_{i = 0}^{n} \alpha_{8i} {\text{EPU}}_{t - i} + \varepsilon_{t} \\ \end{aligned}$$

The short-term ARDL model’s equation (Eq. 3) also called the error correction model is estimated as follows:

3 $$\begin{aligned} {\text{SM}}_{t} = & \, + \mathop \sum \limits_{i = 1}^{m} \alpha_{1i} \Delta {\text{SM}}_{t - i} + \mathop \sum \limits_{i = 0}^{n} \alpha_{2i} \Delta {\text{GDP}}_{t - i} + \mathop \sum \limits_{i = 0}^{n} \alpha_{3i} \Delta {\text{INR}}_{t - i} \\ & \, + \mathop \sum \limits_{i = 0}^{n} \alpha_{4i} \Delta {\text{INF}}_{t - i} + \mathop \sum \limits_{i = 1}^{n} \alpha_{5i} \Delta {\text{UM}}_{t - i} + \mathop \sum \limits_{i = 1}^{n} \alpha_{6i} \Delta {\text{GD}}_{t - i} \\ & \, + \mathop \sum \limits_{i = 1}^{n} \alpha_{7i} \Delta {\text{ER}}_{t - i} + \mathop \sum \limits_{i = 0}^{n} \alpha_{8i} \Delta {\text{EPU}}_{t - i} + \varphi {\text{ECM}}_{t - 1} + \varepsilon_{t} \\ \end{aligned}$$

where $\varphi$ shows the short-term adjustment speed to reach the long-term equilibrium while ${\text{ECM}}_{t - 1}$ is the error correction. This coefficient is anticipated to be significantly negative.

In addition, the Quantile regression and NARDL method have been used to analyze the data and estimate the model. The Quantile regression method was first proposed by Koenker and Bassett [28] and developed in later research. The main reason for using Quantile regression is that it has an accurate view of the response variable and tries to provide a model to allow independent variables to be included not only in the data center of gravity but in all parts of the distribution, especially in the beginning and end sequences. The Quantile regression has many advantages over the ordinary least squares regression (OLS). OLS regression measures the conditional mean of a dependent variable as a function of one or more independent variables, while Quantile regression fully explains the relationship between dependent and independent variables. In other words, OLS regression is a subset of Quantile regression that focuses on the mean [29].

Quantile regression, unlike OLS regression, uses the total absolute value minimization of the weighted residues for estimating the pattern parameters, which is called the absolute minimum value of the deviations [7]. The regression of a multiple of $\theta$ such that $0 < \theta < 1$ is shown as Eq. 4:

4 $$q\left( {\frac{{Y_{t} }}{{{\Omega }_{t} }}} \right) = \theta_{0t} + \theta_{1t} \sum X_{t}$$

In the above relation, $q\left( {\frac{{Y_{{{\text{it}}}} }}{{{\Omega }_{t} }}} \right)$ is a conditional Quantile of the random variable Y, ${\Omega }_{t}$ which contains information about time, and $\sum X_{t}$ is the vector of independent variables affecting the dependent variable. The NARDL method is also has become popular for investigating nonlinear effects in recent years and has been developed by Shin et al. [49]. This method can depict asymmetric and nonlinear aggregation between variables. Can investigate long-term asymmetric and nonlinear effects. The NARDL method is a special form of the linear ARDL format of Pesaran et al. [44], allowing the study of asymmetries in long-term and short-term relationships between variables. The advantage of the NARDL method over other cohesive methods is that it is more efficient in low-observation asset models. NARDL has some advantages: First, this test can be used regardless of whether the model variables are completely *I* (0) and *I* (1) or a combination of both, Second, this method introduces short-term dynamics in the error correction section The third advantage is that this method can be used with a small number of observations and finally it is possible to use this method even when the explanatory variables are endogenous. For this purpose, positive impulses based on Granger and Yoon’s [20] approach are defined as a positive cumulative sum (positive components) and are calculated from the following equation.

5 $$X_{t}^{ + } = \mathop \sum \limits_{t}^{t + 1} \Delta X_{t}^{ + } = {\text{ Max }}(\Delta X. 0)$$

Also, negative shocks based on Granger and Yoon’s (2002) method are defined as a negative cumulative sum (negative components) and are calculated from Eq. 6.

6 $$X_{t}^{ - } = \mathop \sum \limits_{t}^{t + 1} \Delta X_{t}^{ - } = {\text{ Min }}(\Delta X. 0)$$

In the following, after extracting positive and negative impulses, the model will be estimated as follows:

7 $$B_{t} = \mathop \sum \limits_{j = 1}^{p} \varphi_{j} X_{t - j} + \mathop \sum \limits_{j = 0}^{q} (\theta_{j}^{ + } .X_{t - j}^{ + } + \theta_{j}^{ - } .X_{t - j}^{ - } ) + \mathop \sum \limits_{j = 0}^{q} \theta_{j} X_{j} + \varepsilon_{t}$$

where *X* represents the list of explanatory variables affecting the dependent variable *Y*.

## Results and discussion

### Ng: perron unit root test

The Ng-Perron unit root test reflects static variables. Table 3 identifies the findings of the root unit test by intercepting, tracking, and trending. The null hypothesis indicating that SM has a unit root is not rejected at 5% in the model with an intercept and the model with intercept and trend. The initial difference is that the variable appears to be constant, showing that the integration order of the SM variable is one, *I* (1). This situation is the same as other variables used in different models, except for UM which is fixed (Table 3).

**Table 3:** Ng-Perron unit root test

Variables	C						C&T		
	MZ_a_	MZ_t_	MSB	MPT	MZ_a_	MZ_t_	MSB	MPT	S.Breaks
SM	− 1.74	− 0.91	0.52	13.77	− 5.97	− 1.36	0.22	14.77	2008
$\Delta {\text{SM}}$	− 13.32**	− 2.45**	0.18**	2.32**	13.49	− 2.51	18.63	7.20	
GDP	− 1.83	− 0.65	0.35	9.82	− 9.68	− 2.19	0.22	9.43	2008
$\Delta {\text{GDP}}$	− 10.10**	− 2.21**	0.21**	2.53**	− 11.1	− 2.35	0.21	8.20	
INR	− 8.63	− 2.05	0.23	2.90	− 24.2	− 3.43	0.14	4.01*	2001
∆INR	− 11.57**	− 2.33**	0.20**	2.37**	− 11.89	− 2.42	0.20	7.74	
INF	− 7.75	− 1.90	0.24	3.40	− 12.52	− 2.48	0.19	7.36	2009
$\Delta {\text{INF}}$	− 13.50**	− 2.59*	0.192**	1.81**	− 27.85*	− 3.71*	0.13*	3.34*	
UM	− 20.36*	− 3.13*	0.15*	1.40*	− 20.06**	− 3.11**	0.15**	4.85**	2008
$\Delta {\text{UM}}$	− 8.92*	− 2.09**	0.23***	2.80**	− 9.95	− 2.22	0.22	9.16	
GD	1.33	0.68	0.51	24.37	− 4.88	− 1.39	0.28	17.73	1997
$\Delta {\text{GD}}$	− 13.88**	− 2.60*	0.18**	1.87**	− 13.55	− 2.59	0.19	6.77	
ER	− 9.35	− 2.11	0.22	2.81	− 9.16	− 2.10	0.22	10.06	2010
$\Delta {\text{ER}}$	− 12.55**	− 2.48**	0.19**	2.04**	− 12.59	− 2.50	0.19	7.27	
EPU	− 6.50	− 1.52	0.23	4.61	− 9.19	− 2.00	0.21	10.42	2004
$\Delta {\text{EPU}}$	− 13.98*	− 2.56**	0.18**	2.03**	− 13.97	− 2.60	0.18	6.74	

*, **, *** show statistical significance at 1%, 5% and 10% levels, respectively. C and C&T represent constant and constant and trend, respectively. The symbol Δ shows the initial difference between the variables. S. Breaks represent structural breaks in the models with constant only using the Zivot-Andrews unit root test. In Table 3, all of the variables’ structural breaks are statistically significant at 5% levels

Source: Research finding

### ARDL and NRDL models

Table 4 shows the estimation results for both ARDL and NRADL models. As can be seen, in the ARDL model, positive and negative GDP shocks have significant positive effects on SM. This result has also been proved for the NRDL model and in this model, GDP has a positive effect on SM. Negative and positive INR impulses also have a negative effect on SM, but this effect is not significant. There is a similar result in the NRDL model. INF does not have a significant effect on SM in the ARDL model, but in NRDL, INF has a significant negative impact on SM, i.e. with increasing inflation, stock trading decreases. UM negative impulse in the ARDL model has a significant positive impact on SM, but a positive impulse has no significant effect. Also in the NRDL model, UM has a significant negative effect on SM. The positive and negative shocks of GD in the ARDL model have a significant negative impact on SM, which means that with increasing government debt, the amount of stock trading decreases. The effect of GD on SM is not significant in the NARDL model. With increasing positive and negative EPU shocks, the amount of SM in both models decreases quite significantly. That is, increasing economic policy uncertainty is causing the US stock market to stagnate. Therefore, the present study has advised US policymakers to consider the expansionary fiscal policy to increase GDP, consequently, increasing people’s incomes and boosting stock trading and expansionary monetary policy (decreasing the interest rate).

**Table 4:** ARDL and NARDL model results

Variables	NARDL		Variables	ARDL	
	Coefficient	*T* stat		Coefficient	*T* stat
GDP^−^	0.0854**	2.07	GDP	0.640*	28.34
GDP^+^	0.187*	2.24	INR	− 0.217**	6.48
INR^−^	− 0.0157	− 1.72	INR	− 0.034	− 0.74
INR^+^	− 0.048	− 1.68	INF	− 0.215**	− 3.54
INF^−^	− 0.0146	− 1.88	UM	− 0.008**	− 2.55
INF^+^	− 0.0107	− 1.95	GD	0.006	0.06
UM^−^	0.189**	3.36	EPU	− 0.012**	− 3.013
UM^+^	0.046	1.88	–	–	–
GD^−^	− 0.0087*	− 2.29	–	–	–
GD^+^	− 0.0035**	− 2.74	–	–	–
ER^−^	0.0048	1.08	–	–	–
ER^+^	0.0021**	2.96	–	–	–
EPU^−^	− 0.00875**	− 3.68	–	–	–
EPU^+^	− 0.00654**	− 2.07	–	–	–
Bond test	*F* = 7.62			*F* = 10.79	
ECM	− 0.92 (0.000)			− 0.96 (0.000)	
White Heteroscedasticity Test (prob.)	(0.218)			(0.314)	
Breusch–Godfrey serial correlation LM test (prob.)	(0.324)			(0.241)	
Normality test (prob.)	(0.419)			(0.154)	
Ramsey’s test (prob.)	(0.273)			(0.159)	
CUSUM Test	Stable at 5% level			Stable at 5% level	
CUSUM of squares test	Stable at 5% level			Stable at 5% level	
*R*-squared	0.949			0.935	
Adj. *R*-squared	0.901			0.893	
Prob. (*F*-statistic)	0.000			0.000	

*Significant at 1%, **Significant at 5%, and ***Significant at 10%. The values within the () symbols show the probabilities

Source: Research finding

Table 5 provides the terms for correcting the estimated error of the model. As anticipated, error correction terms have significant and negative signs. The estimated error correction is − 0.92 for ARDL and − 0.96 for NARDL, indicating that approximately 92% and 96% of the previous period imbalance will be removed in the current period. To evaluate the diagnosis of the ARDL model, Breusch-Godfrey serial correlation LM test, White heteroscedasticity test, and Ramsey’s RESET test are used. Also, CUSUM and CUSUM of Squares tests evaluate the models’ consistency (Fig. 1). The findings of consistency and diagnostic tests explicitly show that the model lacks unstable parameters, serial correlation, misspecification, and heteroscedasticity.

**Table 5:** Simple Wald test

Variables/Hypothesis tested (C(1)^+^ = C (2)^−^)	Probability
GDP	0.00
INR	0.22
INR	0.47
INF	0.00
UM	0.22
GD	0.22

Source: Research finding

**Fig. 2:**
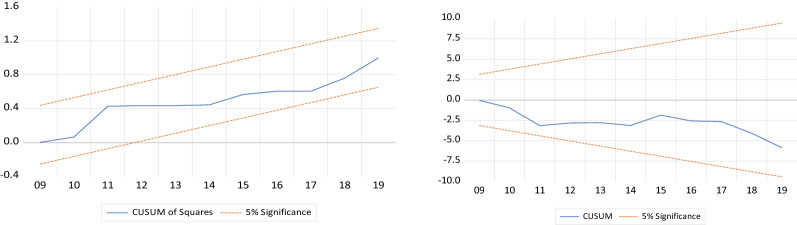
CSUM of Squares and CUSUM. *Source* Research finding

Figure 2 presented the CUSUM and CUSUM of squares tests for the ARDL model. The results detect that the stability is stable at the 5% significance level. Therefore, there are no structural breaks in the US economy in the period 1990–2019.

Furthermore, in the following section, the results of symmetrical or asymmetrical coefficients of different variables on SM are depicted as a criterion for the linearity or nonlinearity of the effects. The null hypothesis of this test, which is based on a simple Wald test, shows that the coefficients are equal and therefore the coefficients are symmetric. The results of this test show that all research variables except INF and GDP were linear with SM. INF and GDP were also nonlinear effects. In other words, the effect of inflation and GDP on SM follows a nonlinear pattern.

### Quantile regression

Table 6 shows the result of the Quantile regression model. Obtained results reflect that the effects of GDP, UM, and ER variables were significant and positive. The effect of the GDP variable on the high quantities of stock trading has also increased. In contrast, the effect of the ER variable on the high quintiles has gradually diminished. Also, the effect of UM variable was positive in low quanta and negative in high quanta. The effect of INR, INF, GD, and EPU variables on income inequality was negative and significant. The effects of INR on high stock trading rates have gradually increased. For the INF variable, the negative effects in the upper quantities have gradually diminished. The effect of the GD variable on stock trading has also gradually diminished in the upper quantities. But for the EPU variable, the negative effects have gradually become greater.

**Table 6:** Quantile regression model

Variables/Quantile	Q20	Q40	Q50	Q60	Q80
GDP	0.087 (7.14)	0.0832 (6.54)	0.0912 (5.16)	0.103 (4.17)	0.108 (4.82)
INR	− 0.105 (− 2.22)	− 0.124 (− 3.71)	− 0.123 (− 3.54)	− 0.120 (− 3.21)	− 0.128 (− 3.95)
INF	− 0.0049 (− 1.83)	− 0.0084 (− 3.46)	− 0.0070 (− 3.07)	− 0.0064 (− 2.63)	− 0.0058 (− 2.17)
UM	0.041 (1.80)	0.028 (1.76)	0.022 (1.89)	− 0.047 (− 1.92)	− 0.058 (− 1.94)
ER	0.159 (4.03)	0.156 (2.19)	0.149 (1.94)	0.148 (2.26)	0.140 (2.53)
GD	− 0.00083 (− 1.78)	− 0.00047 (− 2.02)	− 0.00032 (− 1.25)	− 0.00018 (− 1.5)	− 0.00014 (− 1.55)
EPU	− 0.012 (− 1.78)	− 0.014 (− 1.89)	− 0.019 (− 1.88)	− 0.0247 (− 1.90)	− 0.027 (− 1.91)
C	0.445 (2.88)	0.438 (2.26)	0.458 (2.31)	0.455 (2.11)	0.452 (2.87)

The values within the () symbols show the *T* statistics

Source: Research finding

In the following section, Newey and Powell [38] test (1987) was used to investigate the symmetry of the studied quantiles. The results of Asymmetric Least Squares Estimation and Testing are presented in Table 7. Due to the probability of Newey and Powell’s statistics, the null hypothesis of symmetry of the confirmation results for all variables except GDP and INF is confirmed. In other words, with the increase in stock market transactions, the effect of independent variables (except GDP and INF) has generally increased. The effect of GDP and INF on stock market transactions has also been asymmetric.

**Table 7:** Symmetry results of independent variables

Variables/quantile	0.40–0.60	0.20–0.80
	Prob	Prob
GDP	0.00	0.00
INR	0.133	0.235
INF	0.00	0.00
UM	0.342	0.354
ER	0.198	0.187
GD	0.254	0.246
EPU	0.438	0.464

Source: Research finding

## Conclusion

Forecasting the stock market returns and fluctuations is particularly important for policymakers and portfolio directors. In theory, the returns on assets are functions of the government variables in a real economy. In this regard, there is rich literature that relates microeconomic, macroeconomic, financial, and policy uncertainty indicators to stock returns. The present study aims to investigate the impact of GDP, Inflation Rate, Interest Rate, Economic Policy Uncertainty, Unemployment Rate, Government Debt, and Exchange Rate on Stock Market Trading in the US from 1990 to 2019 using non-ARDL and Quantile regression.

Overall, these findings are complementary to the growing literature regarding the relationship between political-economic uncertainty and stock market transactions. We believe that our results are very important for the debate about the role of political-economic uncertainty in the stock market and fluctuations behavior, and are of great importance to policymakers and international investors who wish to invest in US stock markets. Constant political scandals shake investor confidence and cause unnecessary anxiety and turmoil in financial markets. Therefore, new governments need to restore business confidence to advance financial stability and economic growth in the region.

Based on the results of the NARDL method, the effect of inflation and GDP variables follows a nonlinear pattern. Similar results using quantitative regression showed that the impacts of inflation and GDP on the stock market transactions have been asymmetric. The effects of interest rate variables, unemployment, real effective exchange rates, government debt, and government policy uncertainty on the stock market transactions have also been symmetrical and linear. Therefore, these results advised US policymakers to consider an expansionary fiscal policy to increase GDP, consequently increase people’s incomes and boost stock trading and expansionary monetary policy (decreases the interest rate).

## Data Availability

The data that support the findings of this study are available from the corresponding author, [Bakhtiar Javaheri], upon reasonable request.

## Abbreviations

NARDL: Nonlinear auto-regressive distributed lag
ARDL: Auto-regressive distributed lag
US: Unites State America
EPU: Economic policy uncertainty
GDP: Gross domestic product
